# A large cohort study identifying a novel prognosis prediction model for lung adenocarcinoma through machine learning strategies

**DOI:** 10.1186/s12885-019-6101-7

**Published:** 2019-09-05

**Authors:** Yin Li, Di Ge, Jie Gu, Fengkai Xu, Qiaoliang Zhu, Chunlai Lu

**Affiliations:** Department of Thoracic Surgery, Zhongshan Hospital, Fudan University, 180 Fenglin Road, Shanghai, 200032 People’s Republic of China

**Keywords:** Lung adenocarcinoma, Prognosis prediction model, Machine learning, TCGA, GEO

## Abstract

**Background:**

Predicting lung adenocarcinoma (LUAD) risk is crucial in determining further treatment strategies. Molecular biomarkers may improve risk stratification for LUAD.

**Methods:**

We analyzed the gene expression profiles of LUAD patients from The Cancer Genome Atlas (TCGA) and Gene Expression Omnibus (GEO). We initially used three distinct algorithms (sigFeature, random forest, and univariate Cox regression) to evaluate each gene’s prognostic relevance. Survival related genes were then fitted into the least absolute shrinkage and selection operator (LASSO) model to build a risk prediction model for LUAD. After 100,000 times of calculation and model construction, a 16-gene-based prediction model capable of classifying LUAD patients into high-risk and low-risk groups was successfully built.

**Results:**

Using a combined strategy, we initially identified 2472 significant survival-related genes. Functional enrichment analysis demonstrated these genes’ relevance to tumor initiation and progression. Using the LASSO method, we successfully built a reliable risk prediction model. The risk model was validated in two external sets and an independent set. The expression of these 16 genes was highly correlated with patients’ risk. High-risk group patients witnessed poorer recurrence-free survival (RFS) and overall survival (OS) compared to low-risk group patients. Moreover, stratification analysis and decision curve analysis (DCA) confirmed the independence and potential translational value of this predictive tool. We also built a nomogram comprising risk model and stage to predict OS for LUAD patients.

**Conclusions:**

Our risk model may serve as a practical and reliable prognosis predictive tool for LUAD and could provide novel insights into the understanding of the molecular mechanism of this disease.

**Electronic supplementary material:**

The online version of this article (10.1186/s12885-019-6101-7) contains supplementary material, which is available to authorized users.

## Background

Lung cancer remains the leading cause of cancer death worldwide [1]. The 5-year overall survival rate for lung cancer patients remains low at about 17% [2]. Lung cancer consists of two major histological types: Non-small-cell lung cancer (NSCLC), which accounts for approximately 85%, and small-cell lung cancer (SCLC). Lung adenocarcinoma (LUAD) is the major histological subtype of NSCLC, accounting for more than 40% of lung cancer incidence [3]. For patients with LUAD, early surgical resection is currently the standard treatment. After surgical intervention, patients usually would receive additional chemotherapy, and the survival rate could be improved by 5 to 10% [4]. Despite that, nearly half of LUAD patients still suffered a relapse and would die as a result of disease recurrence [5]. Traditionally, risk factors including tumor size, stage, and lymph node status are commonly used for LUAD patients’ risk assessment and therapeutic plan determination. However, these clinicopathological risk factors fail to clearly distinguish between patients who have a high or low risk and do not predict which patients are more likely to benefit from adjuvant chemotherapy. Therefore, besides traditional clinicopathological risk factors, the discovery of a novel prediction signature which is capable of predicting prognosis for LUAD patients and identifying the high-risk subgroup of LUAD patients is urgently demanded.

In pursuit of predictive biomarkers for patients with LUAD, previous studies had highlighted various biomarkers that may have the potentiality to be used for prognosis prediction in LUAD. However, the limitations of some of these studies included small study populations, lack of validation, single-center cohorts, and model overfitting [6, 7].

Currently, technological advancements in high-throughput techniques such as sequencing and microarray have enabled researchers to examine genetic alterations in carcinogenesis and discovering biomarkers for many diseases [8, 9]. Meanwhile, machine learning methods have been introduced, tuned, and applied into genetic and genomic data to elucidate complex cellular mechanisms, identify molecular signatures, and predict clinical outcomes from large biomedical datasets [10–12].

In this study, we aimed to identify and validate overall survival (OS) related prediction model in LUAD. Different populations of LUAD patients were enrolled in our study. We initially used machine learning algorithms (sigFeature and random forest) and univariate Cox regression analysis to select survival relevant candidate genes in 492 patients from The Cancer Genome Atlas (TCGA) followed by gene signature model construction using LASSO Cox regression analysis in the training set. A 16-gene-based prediction model for LUAD was successfully built after 100,000 times of model construction. We then validated and evaluated the signature classifier from various aspects. We hope that this predictive signature could benefit patients with LUAD and provides more insights into the molecular mechanisms of this prevalent and devastating disease.

## Methods

### Data acquisition and preprocessing

The TCGA LUAD legacy level-3 RNA-Seq data, containing 515 tumor samples and 59 adjacent normal samples, were downloaded, normalized, and quantile filtered using the *TCGAbiolinks* R package [13]. The corresponding clinical information of TCGA LUAD patients was acquired from GDC portal (https://gdc.cancer.gov/about-data/publications/PanCan-Clinical-2018) [14]. Patients with follow-up time less than 30 days were excluded, and finally, a total of 492 TCGA LUAD patients were enrolled in this study as the discovery set.

For GEO data, the database was thoroughly queried for all datasets involving studies of LUAD. To promote the reliability of the results, only datasets supported by peer-reviewed Pubmed-indexed publications, with complete documented clinical survival information of LUAD patients, with sufficient (at least 30) tumor samples, and with accessible raw gene expression profiles, were selected for this study. Based on these criteria, five gene expression microarray datasets (GSE19188, GSE30219, GSE31210, GSE37745, GSE50081) representing different independent studies of LUAD were screened out. After examining the corresponding survival information of each of the five datasets, a total of 579 GEO LUAD patients with follow-up time longer than 30 days were included in this study as external sets.

The gene expression profiles for all five datasets were generated from the Affymetrix Human Genome U133 Plus 2.0 Array. The raw CEL files of 579 GEO LUAD patients were downloaded from the repository and were uniformly processed using the Robust Multichip Average (RMA) algorithm for background correction and normalization. The R package *affy* was chosen as the implementation of this algorithm [15].

The probe sets of Affymetrix HG-U133 Plus 2.0 Array were annotated to genes based on the annotation platform GPL570. For each gene, all corresponding probe set signals were averaged to produce a single expression value. Finally, the expression data of 21,755 genes was obtained. Next, the batch correction was performed, followed by normalization between arrays to remove the heterogeneity among multiple microarray datasets using *sva* and *limma* packages (Additional file 1: Figure S1) [16, 17].

Apart from the above cohorts, we also downloaded another dataset (GSE72094) as an independent set for further validation using *GEOquery* R package.

### Candidate genes selection using three distinct algorithms

We used the discovery set (492 TCGA LUAD patients) to select candidate genes in this step. We examined each gene’s prognosis relevance using three different methods (sigFeature, random forest, and univariate Cox regression).

SigFeature algorithm is a combined machine learning approach which is capable of identifying the significant features using support vector machine recursive feature elimination method (SVM-RFE) and t-statistic [18, 19]. In this study, we used sigFeature method to rank all the genes based on their discriminative power of distinguishing alive patients from dead patients and selected top 1000 genes for subsequent analysis. The R package *sigFeature* was used as the implementation of this algorithm (http://bioconductor.org/packages/release/bioc/html/sigFeature.html).

The random forest algorithm is also a machine learning strategy, which is based on the construction of many classification (decision) trees that are used to classify the input data vector [20]. *RandomForestSRC* package is an extension of the original random forest method and supports models including survival, regression, and classification. Using this method, a total of 892 genes were considered important survival relevant variables for subsequent analysis (https://kogalur.github.io/randomForestSRC/).

Univariate Cox regression analysis is also a popular method for determining the potential prognostic factors. In this study, the independent hazard rate for each gene was calculated based on the discovery set, and a descriptive *p*-value of < 0.005 was considered statistically significant. The R package *survival* was used as the implementation of this method.

### Functional annotation

Functional enrichment analysis (FEA) was used to confirm the biological relevance of the genes identified from the methods above. The R package *MoonlightR* was used to perform this analysis [21].

### Predictive models construction and selection

After obtaining the candidate genes from the methods above, we used the LASSO Cox regression analysis to select the most significant prognostic genes in training set for predictive model construction. LASSO is a penalized strategy that is suitable for high-dimensional data and can prevent overfitting [22, 23]. Here, we used 10 folds cross-validation to determine the values of λ, and we chose the λ where the partial likelihood deviance is the smallest as the optimal λ. Once the predictive genes were determined, we applied them to build an expression-based risk model by risk score method as follows:

$$ Risk\ Score=\sum \limits_{i=1}^N\left({Exp}_i\ast {C}_i\right) $$

Where *N* is the number of genes, *Exp*_*i*_ is the expression level of *gene*_*i*_, and *C*_*i*_ is the coefficient of *gene*_*i*_ obtained from the LASSO Cox regression analysis in the training set. We calculated the concordance index (C-index) to evaluate the predictive accuracy of the risk model preliminarily.

We then used computer-generated random numbers to divide 492 TCGA LUAD patients into the training (345 cases) and internal testing (147 cases) sets. The training models were then applied to the internal testing set, the entire TCGA set, the external testing set (232 patients from GSE37745 and GSE50081) and the external validation set (347 patients from GES19188, GSE30219, and GSE31219) for the optimal model selection. Here, we considered a model whose C-index greater than 0.680 in every LUAD patient cohort to be reliable and stable. R packages *glment* and *Hmisc* were used as the implementation of this method.

### Statistical analysis

After 100,000 times of model construction, a well-performed and stable 16-gene-based prognosis prediction model outstood, with which every LUAD patient was assigned a risk score, and LUAD patients were divided into high-risk and low-risk groups according to the optimal cut-off value (minimum *p*-value) of the risk score. We then performed the time-dependent receiver operating characteristic (ROC) analysis and calculated area under the curve (AUC) at different cut-off times to measure the discriminative accuracy of this particular model. The R package *survminer* and *survivalROC* were used to calculate the best cut-off value and performed ROC analysis, respectively.

To further discover whether this model has advantages over other commonly used clinical parameters, and is worth using in clinical practice, decision curve analysis (DCA) was performed to evaluate the predictive model [24]. The R code for DCA is available at http://www.decisioncurveanalysis.org along with tutorials.

Next, we did a multivariate Cox regression, and the coefficients of the multivariable Cox regression model were used to construct a nomogram with the *rms* package. The performance of the nomogram was assessed by the C-index via a bootstrap method and was visualized by calibration plots.

To explore the potential biological relevance of the prediction signature, gene set enrichment analysis (GSEA) was performed using R package *clusterProfiler* to rank gene sets associated with risk [25]. The Reactome gene sets (http://software.broadinstitute.org/gsea/msigdb/genesets.jsp?collection=CP:REACTOME) containing 674 gene sets were downloaded from MSigDB [26]. The gene sets with positive enrichment score (or negative enrichment score) and *p*-value < 0.05 after 1000 permutations were considered significantly enriched gene sets.

## Results

### Patient characteristics

**Fig. 1:**
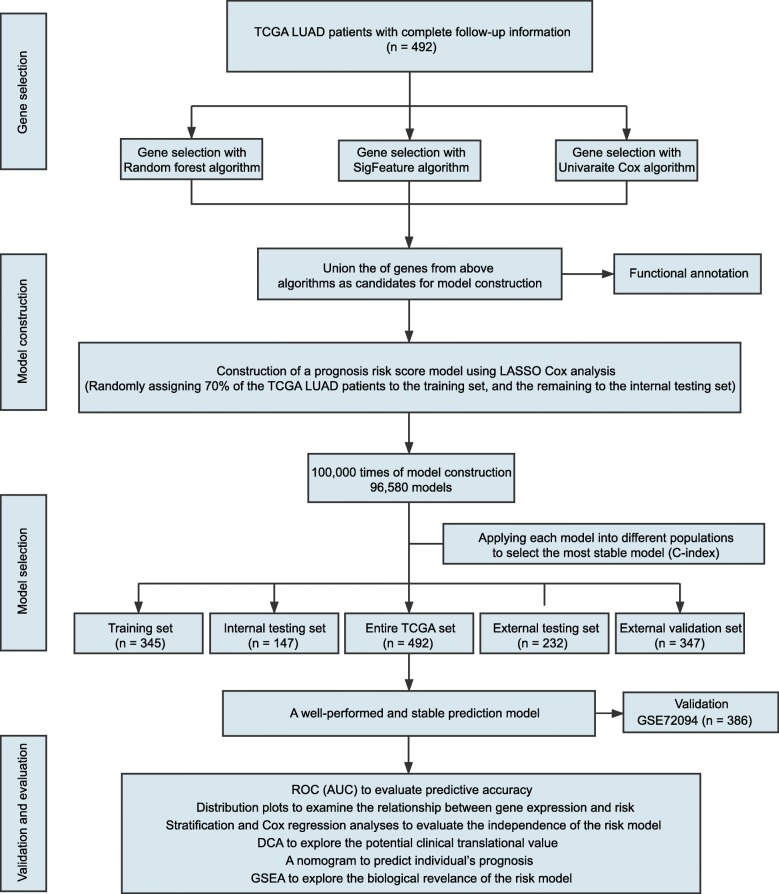
Study flow chart for our analysis. TCGA: The Cancer Genome Atlas; LUAD: Lung Adenocarcinoma; ROC: Receiver Operating Characteristic (ROC) analysis; AUC: Area Under the Curve; DCA: Decision Curve Analysis; GSEA; Gene Set Enrichment Analysis

The study flowchart is illustrated in Fig. 1. Common clinical characteristics of these patients were summarized in Table 1. A total of 1463 LUAD patients were enrolled in our study, among which 492 patients were assigned to the discovery set, 232 patients were assigned to the external testing set, 347 patients were included as the external validation set, and 386 patients were assigned to the independent set. The median OS time of patients in the discovery set, external testing set, external validation set, and independent set were 667.5 days (IQR 432.8–1147.2), 1551.25 days (IQR 594.0–2294.9), 1803.0 days (IQR 1125.0–2391.0) and 831.5 days (IQR 568.5–1022.8), respectively. One hundred seventy-eight patients in the discovery set, 127 patients in the external testing set, 100 patients in the external validation set, and 109 patients in the independent set were deceased during follow-up. Detailed clinicopathological features of these patients were shown in Additional file 2: Table S1.

**Table 1:**
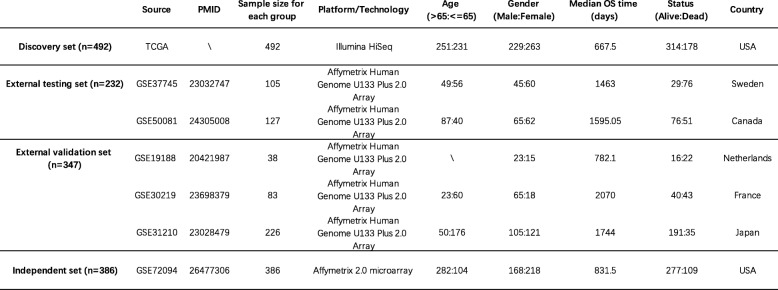
Clinical characteristics of LUAD studies from TCGA, GEO and ICGC data repositories

### Genes determined by three algorithms

**Fig. 2:**
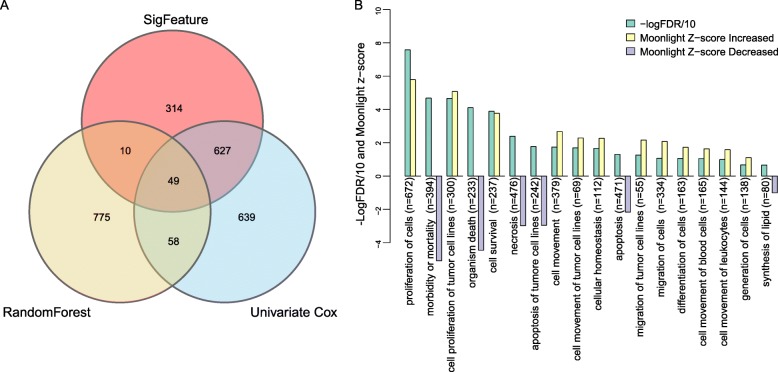
Survival-related genes selected by the three algorithms from the discovery set and functional annotation. **a** 1000 genes from sigFeature algorithm, 892 genes from random forest algorithm and 1373 genes from univariate Cox algorithm. There are 2472 genes in total, and 49 genes that are in the overlapping region of the three algorithms. **b** Top 18 biological processes enriched significantly with |Moonlight-score| > =1 and FDR < 0.05 using above 2472 genes. Increased activities highlighted in yellow and decreased in purple, green indicates the -Log_10_FDR. A negative z-score indicates the activity of this biological process is decreased and a positive z-score indicates the opposite

Three different algorithms, i.e., sigFeature, random forest, and univariate Cox, were used to select the survival-relevant genes before model construction, and we hypothesized that the combination of the genes identified by each of the three algorithms was more survival-related and therefore more suitable for prognosis prediction for LUAD patients. A total of 2472 genes were identified (1000 genes from sigFeature algorithm, 892 genes from random forest algorithm and 1373 genes from univariate Cox regression analysis), with 49 genes selected simultaneously by the three algorithms (Fig. 2a and Additional file 3: Table S2). Functional enrichment analysis was performed on these 2472 genes, and we found that expression alterations of these genes could activate tumor progression-related biological processes such as proliferation of cells, cell proliferation of tumor cell lines, cell survival, cell movement of tumor cell lines, migration of tumor cell lines and cell movement of blood cells and leukocytes, and deactivate processes including morbidity or mortality, organism death, necrosis, apoptosis of tumor cell lines and synthesis of lipid (Fig. 2b and Additional file 4: Table S3, |Z-score| > 1, *p*-value < 0.05).

### Building a predictive model for LUAD

**Fig. 3:**
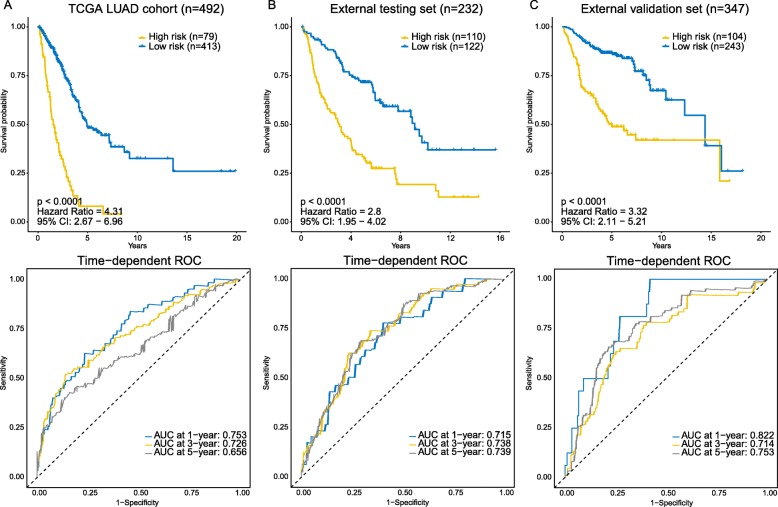
Kaplan-Meier survival analysis and time-dependent ROC curves in the TCGA cohort, external testing, and external validation sets. AUC: area under the curve. **a** TCGA LUAD cohort. **b** External testing set. **c** External validation set. We used AUCs at 1, 3, and 5 years to assess prediction accuracy, and calculated *p*-values using the log-rank test

**Fig. 4:**
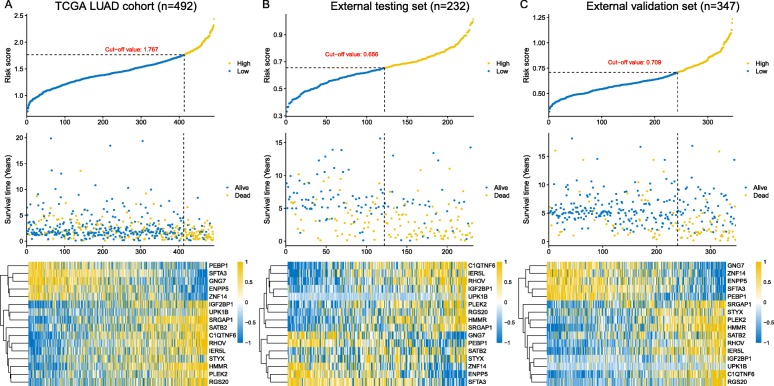
The distribution of risk scores, patients’ survival status and the heatmap of gene expression profiles in the TCGA cohort, external testing, and external validation sets. **a** TCGA LUAD cohort. **b** External testing set. **c** External validation set

In order to build a clinically available risk prediction model for different populations of LUAD patients, expression data of the 2472 genes in 345 patients from the training set were subjected to the LASSO Cox regression analysis. In the initial 50,000 times of model construction, we failed to build a reliable and stable risk model that could be validated in different populations of LUAD patients. On the 54,360th trial, we successively captured a well-performed and stable prediction model, with C-indices reaching 0.700, 0.689, 0.696, 0.682, and 0.704 in the training set, internal testing set, entire TCGA set, external testing set, and external validation set, respectively (Additional file 1: Figure S2). We then continued to increase the number of trials to see whether there would be a better risk model. However, as we continued to increase the number of trials, the performance of models tended to level off. After 100,000 times of calculation, a total of 96,580 prediction model consisting of various gene signatures were constructed, and we did not find another better model (Additional file 5: Table S4). Finally, 16 critical prognostic genes were successfully extracted. Based on the coefficients generated from the LASSO Cox regression analysis, an expression-based risk score model was built using the formula described in the Materials and methods section (Additional file 6: Table S5). Using above risk score model, each patient of the TCGA LUAD cohort was assigned a risk score according to expression values of 16 gene biomarkers, and then patients were separated into high-risk and low-risk groups using the optimal cut-off value (1.767) (Fig. 4a, top panel). Seventy-nine high-risk LUAD patients had poorer OS (hazard ratio [HR], 4.31; 95% CI, 2.67 to 6.96; *p*-value < 0.0001) than did the 413 low-risk LUAD patients (Fig. 3a, top panel). We further assessed the prognostic accuracy of the 16-gene-based classifier with time-dependent ROC analysis at varying follow-up times (Fig. 3a, bottom panel), and the area under the curve (AUC) received 0.753, 0.726 and 0,656 at 1-, 3- and 5-year. We also assessed the distribution of the risk score, survival status and expression patterns of the 16-gene classifier in the TCGA LUAD cohort, patients with lower risk scores generally had better outcomes than those with higher risk scores, and the former tended to have higher expression of PEBP1, SFTA3, GNG7, ENPP5 and ZNF14, whereas the latter tended to have higher expression of the remaining genes (Fig. 4a, middle and bottom panels).

### Evaluating the prediction model

To further substantiate the availability and stability of this 16-gene-based risk model, we did the same analyses on the two external sets (Additional file 7: Table S6). For the external testing set (*n* = 232), the optimal cut-off value for classifying LUAD patients into high- and low-risk group was 0.656 (Fig. 4b, top panel), with which the model successfully categorized 110 patients into the high-risk group and 122 patients into the low-risk group, which were significantly different in terms of OS (HR, 2.8; 95% CI, 1.95 to 4.02; *p*-value < 0.0001; Fig. 3b, top panel). The time-dependent ROC analysis suggested the AUC was 0.715, 0.738, and 0.739 at 1-, 3- and 5-year for this cohort (Fig. 3b, bottom panel). Likewise, validation on the external validation set (*n* = 347) showed consistent result that high-risk group patients (*n* = 104) had poorer OS compared to low-risk group patients (*n* = 243) (HR, 3.32; 95% CI, 2.11 to 5.21; *p*-value < 0.0001; Fig. 3c, top panel). The AUC was 0.822, 0.714, 0.753 at 1-, 3- and 5-year (Fig. 3c, bottom panel).

The distribution of the risk score, survival status, and expression patterns of the 16-gene classifier in two external sets also showed consistent results with the TCGA LUAD cohort. Higher risk score patients had poor survival than lower risk score patients, and the former tended to have over-expression of IGF2BP1, UPK1B, SRGAP1, SATB2, C1QTNF6, RHOV, IER5L, STYX, HMMR, PLEK2, RGS20 and lower expression of PEBP1, SFTA3, GNG7, ENPP5 and ZNF14 (Fig. 4b and c).

On the other hand, in order to further demonstrate that this predictive signature also works on other cohorts, we further included another independent LUAD cohort (*n* = 386) from GSE72094 dataset. In this independent cohort, the predictive tool is also able to classify LUAD patients into high- and low-risk groups with different survival outcomes (Additional file 1: Figure S6, *p*-value < 0.0001). Both univariate (HR: 2.617; 95%CI: 1.785–3.833; C-index: 0.663; *p*-value < 0.0001) and multivariate (HR: 2.360; 95% CI: 1.603–3.472; *p*-value < 0.0001) Cox regression analyses of the risk-score model on this cohort showed that this tool is also an independent prognostic indicator.

**Fig. 5:**
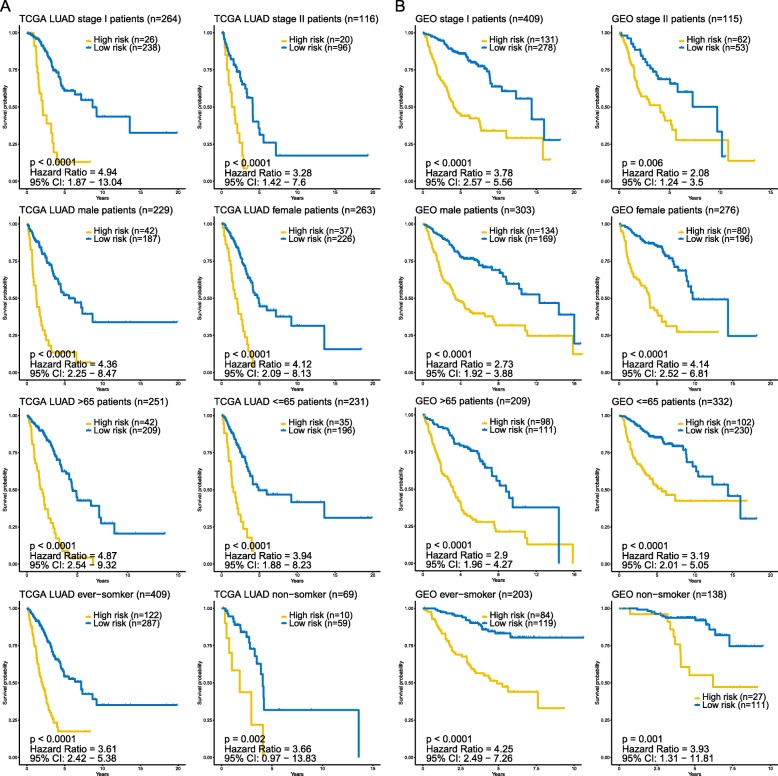
Kaplan-Meier survival analysis for all TCGA LUAD patients and GEO LUAD patients according to the 16-gene-based model stratified by clinical stage, gender, age, and smoking status. **a** TCGA LUAD patients. **b** GEO LUAD patients

To determine whether the prognostic value of the predictive signature was independent of other clinicopathological variables of the patients with LUAD, both univariate and multivariate Cox regression analysis were performed. Selected variables included age, gender, stage, smoking, and our risk model. The results of univariate and multivariate Cox regression analysis from TCGA and GEO patient datasets demonstrated that this predictive risk model was an independent prognostic factor for LUAD patients after adjusted by these clinical variables (Table 2). Besides, age and stage were also found significant in both LUAD patient datasets. In order to further validate whether this model could apply to different groups of LUAD patients in terms of clinical variables (clinical stage, gender, and age), we also did a stratification analysis on TCGA LUAD cohort and entire GEO LUAD cohort, respectively. The stratification analysis was first carried out in tumor stage, which stratified patients into the stage I group and stage II group. For patients within stage I group, both TCGA and GEO patient cohorts witnessed significant differences of OS between high- and low-risk group (Fig. 5). As to the stage II group, OS was also significantly different between the two risk groups. Since the number of stage III patients in the GEO cohort and IV patients in both cohorts were limited, the stratification analyses on these subgroups were not conducted (Additional file 1: Figure S4). Stratification analyses on gender, age, and smoking also showed consistent results that in different groups of LUAD patients, this predictive signature was still capable of classifying patients into high- and low-risk groups, which were significantly different in terms of OS. Taken together, the results of Cox regression and stratification analysis suggested that the predictive signature is independent of other clinical features for prognosis prediction of LUAD patients.

**Table 2:**
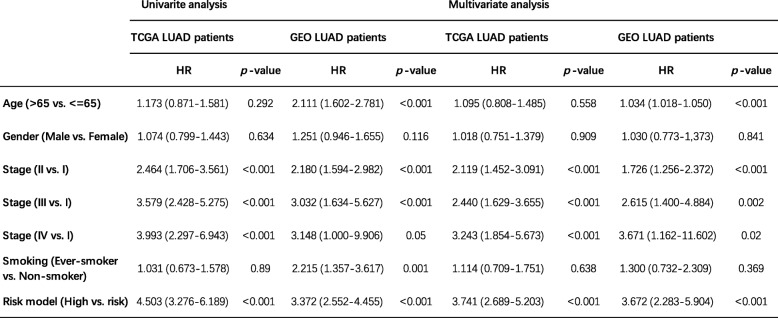
Univariate and multivariate analyses of clinicopathological factors and risk model in TCGA and GEO LUAD cohorts

Apart from predicting OS, this predictive signature was also able to predict recurrence in LUAD patients. We observed that high-risk group patients had shorter recurrence-free survival (RFS) compared to low-risk group patients in both TCGA (HR, 2.38; 95% CI, 1.13 to 5.01; *p*-value = 0.001) and GEO (HR, 2.94; 95% CI, 2.06 to 4.2; *p*-value < 0.0001) LUAD patients (Additional file 1: Figure S5).

**Fig. 6:**
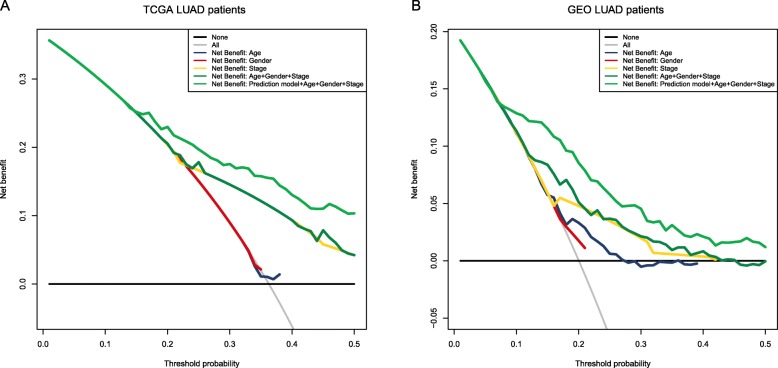
Decision curve analysis for the risk prediction model. **a** TCGA LUAD patients. **b** GEO LUAD patients. Black line: assume no patient is at high-risk. Grey line: assume all patients are at high-risk. These two lines serve as a reference. Light green line: adding the 16-gene-based model to clinicopathological risk factors can provide more net benefits for LUAD patients’ survival prediction

After evaluating the prediction accuracy and independence of this model, we focused on whether the application of these model plus common in-use clinical parameters could benefit LUAD patients in clinical practice. We did DCA on our prediction model to assess the net benefit that patients could receive. As is shown in Fig. 6, for both TCGA LUAD patients and GEO LUAD patients, they could gain more benefits when we combined our prediction model to age, gender and stage in predicting prognosis (Fig. 6).

These results indicated that our 16-gene-based prediction model performed well and was capable of distinguishing different populations of LUAD patients with high or low risk of survival.

### Building a nomogram for individual patient’s prognosis prediction

**Fig. 7:**
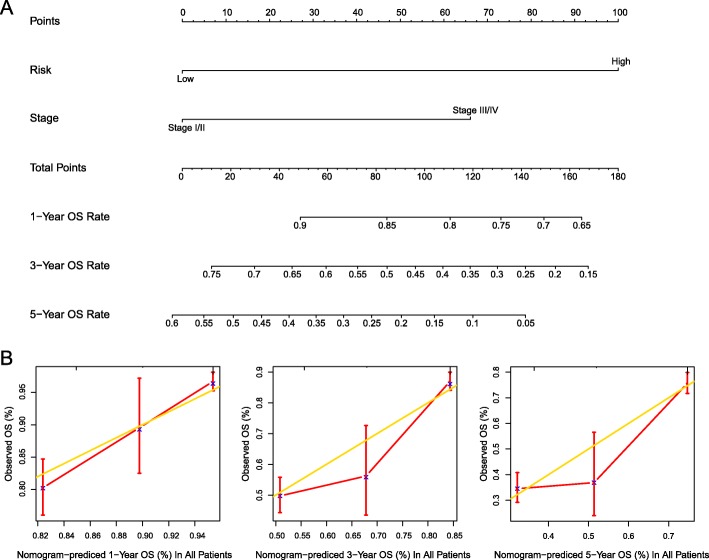
Nomogram to predict OS for individual LUAD patient. **a** Nomogram predicting 1-, 3-, 5-year OS rate. **b** Calibration curves showing predicted OS rate vs. observed OS rate. The gold line represents the ideal OS rate, and the red line represents the observed OS rate

To develop a clinically applicable method that could predict an individual’s OS probability, we used a nomogram to build a predictive model. The nomogram was generated on the basis of the multivariate analysis (*p*-value < 0.05) of OS in the TCGA LUAD patients (Fig. 7a). The calibration plots for the 1-, 3-, 5-year OS rate were predicted well in entire LUAD patients (C-index: 0.695 for 1-year, 0.694 for 3-year and 0.695 for 5-year; Fig. 7b).

### Identification of gene signature-related biological functions using GSEA

**Fig. 8:**
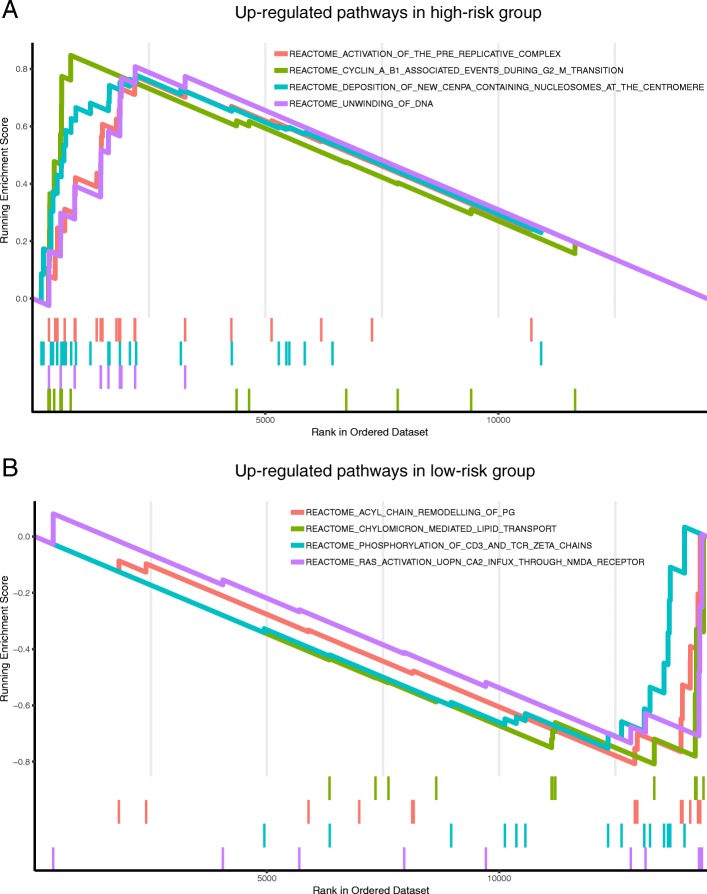
Gene set enrichment analysis associated with risk using the Reactome database. **a** Top enriched biological pathways in high-risk group patients. **b** Top enriched biological pathways in low-risk group patients. (*p*-value < 0.05)

In order to gain more insights into the biological functions of the risk prediction signature, we applied GSEA to identify associated biological pathways from gene expression profiles of LUAD patients in the high-risk and low-risk groups classified by the gene signature. The high-risk group patients were associated with multiple up-regulated gene sets, mainly involved in activation of the pre-replicative complex, cyclin A/B1 associated events during G2/M transition, deposition of new CENPA-containing nucleosomes at the centromere and unwinding of DNA. On the other hand, the low-risk group patients were associated with up-regulation of acyl chain remodeling of PG, chylomicron-mediated lipid transport, phosphorylation of CD3 and TCR zeta chains and Ras activation upon Ca2+ influx through NMDA receptor (Fig. 8 and Additional file 8: Table S7, *p*-value < 0.05).

## Discussion

Previous studies have demonstrated many different single prognostic biomarkers for LUAD. SOX30 can inhibit tumor-metastasis by directly binding to CTNNB1 promoter and result in a favorable prognosis [27]. Elevated expression of lncRNA-ATB suggests a poor prognosis of NSCLC and leads to cell proliferation and metastasis in NSCLC [28]. Besides, MicroRNA-30e-5p is found to be over-expressed in LUAD, associating with tumor size and tumor progression [29]. However, these biomarkers cannot separate high-risk patients from low-risk patients with LUAD. On the basis of single prognostic biomarkers, integrating multiple biomarkers into a single prediction model would practically promote prognostic value compared with a single biomarker [12, 30, 31]. However, several limitations of early studies with integrated models cannot be neglected. (1) There were insufficient number of patients, which could lead to model overfitting [32]. (2) Models were not validated in independent cohorts [33, 34]. In this study, we used a novel combination strategy that incorporated genes from three distinct algorithms (i.e., two novel machine learning methods: sigFeature and random forest, and a traditional univariate Cox regression) to minimize the possibility of losing or ignoring important survival-related biomarkers [35]. The FEA of the genes selected from the three distinct algorithms demonstrated that these genes could activate tumor progression-related biological processes such as proliferation of cells, cell proliferation of tumor cell lines, cell survival, cell movement of tumor cell lines, migration of tumor cell lines and cell movement of blood cells and leukocytes, and down-regulate biological processes including morbidity or mortality, organism death, necrosis and apoptosis of tumor cell lines. Increased activities of proliferation, movement, and migration of tumor cells and decreased activities of apoptosis can lead to tumor recurrence, progression, and metastasis [36]. Immune infiltrations in the blood are also associated with recurrence and tumor progression [37]. Fatty acid synthesis exhibited multifaceted roles in cancer [38]. Tumor cells must overcome these various forms of cell death to metastasize. Therefore, the results from FEA have confirmed the feasibility of our combination strategy.

Integrating multiple studies of a particular disease can significantly increase the number of samples and has been shown to improve the detecting power [39, 40]. In this study, we integrated the expression profiles of LUAD from TCGA and GEO data repositories. A total of 1463 patients with LUAD were enrolled. Using the LASSO method, we established a 16-gene-based prediction model for LUAD. This novel prognostic signature was successfully validated in the internal testing set, the entire TCGA cohort, two external cohorts and an independent cohort, which indicated the stability and robust discriminative power of this signature in terms of classifying LUAD patients into high- and low-risk subgroups.

Using this prognostic signature, LUAD patients could be stratified into high- and low-risk subgroups. Generally speaking, high-risk patients should receive more frequent clinical surveillance and corresponding measures to prevent disease recurrence and progression [41]. In our study, we observed that high-risk subgroup had higher risks of recurrence and poor clinical outcomes compared to the low-risk subgroup. Therefore, our predictive signature may help identify high-risk LUAD patients and make appropriate clinical follow-up plans accordingly.

The conventional indicators for predicting prognosis and making additional adjuvant treatment decisions for LUAD patients after surgical resection include tumor stage, tumor size, lymph node status, age, and so on [42]. To assess the independence of the predictive signature in prognosis prediction, we performed both univariate and multivariate Cox regression analysis. In both TCGA and GEO LUAD patients, the predictive signature maintained an independent correlation with OS after adjusting for age, gender, stage, and smoking. In the stratification analysis, the predictive signature classified patients within the same age stratum, the same gender, the same stage, and the smoking status into high- and low-risk subgroups. The results showed that patients in the high-risk group tended to have shorter OS than those in the low-risk group across these stratified patient datasets, indicating the age-, gender-, stage-, and smoking-independent value of the predictive signature.

In order to evaluate the prediction accuracy of the predictive signature, we did the time-dependent ROC analysis and calculated AUCs at different cut-off times. The AUCs received 0.753, 0.726 and 0,656 at 1-, 3- and 5-year in the TCGA cohort, 0.715, 0.738 and 0.739 at 1-, 3- and 5-year in the external test set and 0.822, 0.714, 0.753 at 1-, 3- and 5-year in the external validation set, suggesting relatively ideal predictive accuracy. However, the AUC focuses merely on the predictive accuracy of the signature. As such, it does not tell us whether the model is worth using at all. DCA is a statistical method that incorporate consequences and, thus, can inform the decision of whether to use this model [24]. By applying the method to our predictive signature along with clinical factors, age, gender, and stage on both TCGA and GEO patients, we found that LUAD patients benefitted more when combining the predictive signature with the above clinical factors to predict prognosis. In addition, we also built a nomogram including stage and our risk model to predict individual prognosis. The performance of the nomogram was validated in the entire cohort. Thus, this nomogram may provide an accurate prognosis prediction for LUAD.

As to the prediction model itself, we found patients with high-risk scores tended to have higher expression of IGF2BP1, UPK1B, SRGAP1, SATB2, C1QTNF6, RHOV, IER5L, STYX, HMMR, PLEK2, and RGS20, and low-risk scores patients tended to be positively correlated with PEBP1, SFTA3, GNG7, ENPP5, and ZNF14. IGF2BP1 is an RNA-binding protein predominantly involving in tumor progression, and its expression is associated with poor prognosis in cancers [43, 44]. SATB2 is involved in the progression of breast cancer, head and neck squamous cell carcinomas, and osteosarcoma [45]. PEBP1, an RAF kinase inhibitory protein, is involved in lipid death signals, and negatively regulates starvation-induced autophagy [46, 47]. However, the roles of UPK1B, SRGAP1, C1QTNF6, RHOV, IER5L, STYX, HMMR, PLEK2, RGS20, SFTA3, GNG7, ENPP5, and ZNF14 in LUAD initiation and progression are still not well understood.

In eukaryotes, DNA replication is mediated by the assembly of the pre-replicative complex (pre- RC) on replication origins [48]. Cyclin A/B1 during G2/M transition is also a critical regulator of proper DNA replication. CENPA is a critical factor for the assembly of a macromolecular protein complex at the kinetochore [49]. Cancer cell proliferation requires the rapid synthesis of lipids for the generation of biological membranes [50]. In our present study, GSEA found that high-risk group patients were mainly involved in activation of the pre-replicative complex, cyclin A/B1, and CENPA at the centromere and unwinding of DNA. The low-risk group patients were associated with chylomicron-mediated lipid transport, phosphorylation of CD3 and TCR zeta chains and Ras activation upon Ca2^+^ influx through NMDA receptor.

## Conclusion

In summary, we integrated the expression profiles of LUAD from multiple centers based on TCGA and GEO data repositories, and thereby, our results could avoid inherent biases of such a study format. We used a novel combination strategy that incorporated genes from three distinct methods to identify survival-related genes. We developed a 16-gene-based risk model for LUAD prognosis prediction through a comprehensive analysis of different populations of LUAD patients. Moreover, our study showed that this predictive model is effective to classify patients with LUAD into high- and low-risk group. Thus, this predictive tool may help facilitate the development of individualized treatment for LUAD patients.

## Additional files

Additional file 1: **Figure S1.** Normalization of the microarray datasets from GEO. A. Before normalization. The batch effect among five datasets can be observed. B. After removal of batch effect and normalization. **Figure S2.** Model construction and selection. X-axis represents the number of risk models, y-axis represents the C-index, each green point represents the C-index of a particular risk model. We used red lines to connect these points. The C-indices of the model we captured were highlighted. **Figure S3.** 16 genes were selected by LASSO regression analysis. A. The dashed vertical line represents the optimal value of log λ with the minimum partial likelihood deviance. B. LASSO coefficient of the 16 genes. **Figure S4.** Stratification analysis. TCGA stage III LUAD patients were stratified into high- and low- subgroups based the predictive signature, and high-risk group patients had poorer OS compared to low-risk group patients. Hazard Ratio: 2.89; 95% CI: 1.47 − 5.69; *p*-value < 0.001. **Figure S5.** Kaplan-Meier survival curves of recurrence-free survival (RFS) between high-risk and low-risk patients. A. TCGA LUAD patients. B. GEO LUAD patients. In both patient datasets, the RFS time of patients in the high-risk group was significantly shorter than that in the low-risk group. **Figure S6.** Independent validation on GSE72094 dataset. Kaplan-Meier survival analysis showing the predictive signature can separate GSE72094 LUAD patients into high- and low-risk groups with different OS (*p*-value < 0.0001). (DOCX 3130 kb)

Additional file 2: **Table S1.** Clinical information of LUAD patients from TCGA and GEO data repositories (XLSX 269 kb)

Additional file 3: **Table S2.** Gene candidates selected from three algorithms (XLSX 108 kb)

Additional file 4: **Table S3.** Significantly enriched biological processes from functional enrichment analysis (FEA) (XLSX 18 kb)

Additional file 5: **Table S4.** C-index of each set during each loop (Optimal model: loop #54360) (XLSX 5797 kb)

Additional file 6: **Table S5.** LASSO coefficient (XLSX 9 kb)

Additional file 7: **Table S6.** Stratification of LUAD patients based on the predictive model (XLSX 25 kb)

Additional file 8: **Table S7.** Results from GSEA based on Reactome database (*p*-value < 0.05) (XLSX 27 kb)

## Data Availability

All data used in this study can be downloaded from TCGA data repository (https://gdc.cancer.gov) and GEO data repository (https://www.ncbi.nlm.nih.gov/geo).

## Abbreviations

AUC: Area Under the Curve
DCA: Decision Curve Analysis
FEA: Functional Enrichment Analysis
GEO: Gene Expression Omnibus
LUAD: Lung Adenocarcinoma
NSCLC: Non-small-cell Lung Cancer
OS: Overall Survival
RFS: Recurrent Free survival
ROC: Receiver Operating Characteristic analysis
SVM-RFE: Support Vector Machine Recursive Feature Elimination
TCGA: The Cancer Genome Atlas
