# Supplementary material for: A large cohort study identifying a novel prognosis prediction model for lung adenocarcinoma through machine learning strategies
Source: BMC Cancer. 2019 Sep 5;19:886. doi: 10.1186/s12885-019-6101-7 (PMC6729062; doi:10.1186/s12885-019-6101-7)
Supplement: Supplementary file 1 — Figure S1. Normalization of the microarray datasets from GEO. A. Before normalization. The batch effect among five datasets can be observed. B. After removal of batch effect and normalization. Figure S2. Model construction and selection. X-axis represents the number of risk models, y-axis represents the C-index, each green point represents the C-index of a particular risk model. We used red lines to connect these points. The C-indices of the model we captured were highlighted. Figure S3. 16 genes were selected by LASSO regression analysis. A. The dashed vertical line represents the optimal value of log λ with the minimum partial likelihood deviance. B. LASSO coefficient of the 16 genes. Figure S4. Stratification analysis. TCGA stage III LUAD patients were stratified into high- and low- subgroups based the predictive signature, and high-risk group patients had poorer OS compared to low-risk group patients. Hazard Ratio: 2.89; 95% CI: 1.47 − 5.69; p-value < 0.001. Figure S5. Kaplan-Meier survival curves of recurrence-free survival (RFS) between high-risk and low-risk patients. A. TCGA LUAD patients. B. GEO LUAD patients. In both patient datasets, the RFS time of patients in the high-risk group was significantly shorter than that in the low-risk group. Figure S6. Independent validation on GSE72094 dataset. Kaplan-Meier survival analysis showing the predictive signature can separate GSE72094 LUAD patients into high- and low-risk groups with different OS (p-value < 0.0001). (DOCX 3130 kb) [file 12885_2019_6101_MOESM1_ESM.docx]

**Supplementary information**

**A large cohort study identifying a novel prognosis prediction model for lung adenocarcinoma through machine learning strategies**

Yin Li, Di Ge, Jie Gu, Fengkai Xu, Qiaoliang Zhu and Chunlai Lu^*^

Department of Thoracic Surgery, Zhongshan Hospital, Fudan University, Shanghai, P. R. China.

^*^Corresponding author: Chunlai Lu, M.D, Department of Thoracic Surgery, Zhongshan Hospital, Fudan University, 180 Fenglin Road, Shanghai, 200032, P.R.China, Tel. /Fax: +86 021 64041990-2559, E-mail address: lu.chunlai@zs-hospital.sh.cn.

**Running title**: A novel prognosis prediction model for lung adenocarcinoma.

**Figure S1.** Normalization of the microarray datasets from GEO. A. Before normalization. The batch effect among five datasets can be observed. B. After removal of batch effect and normalization.


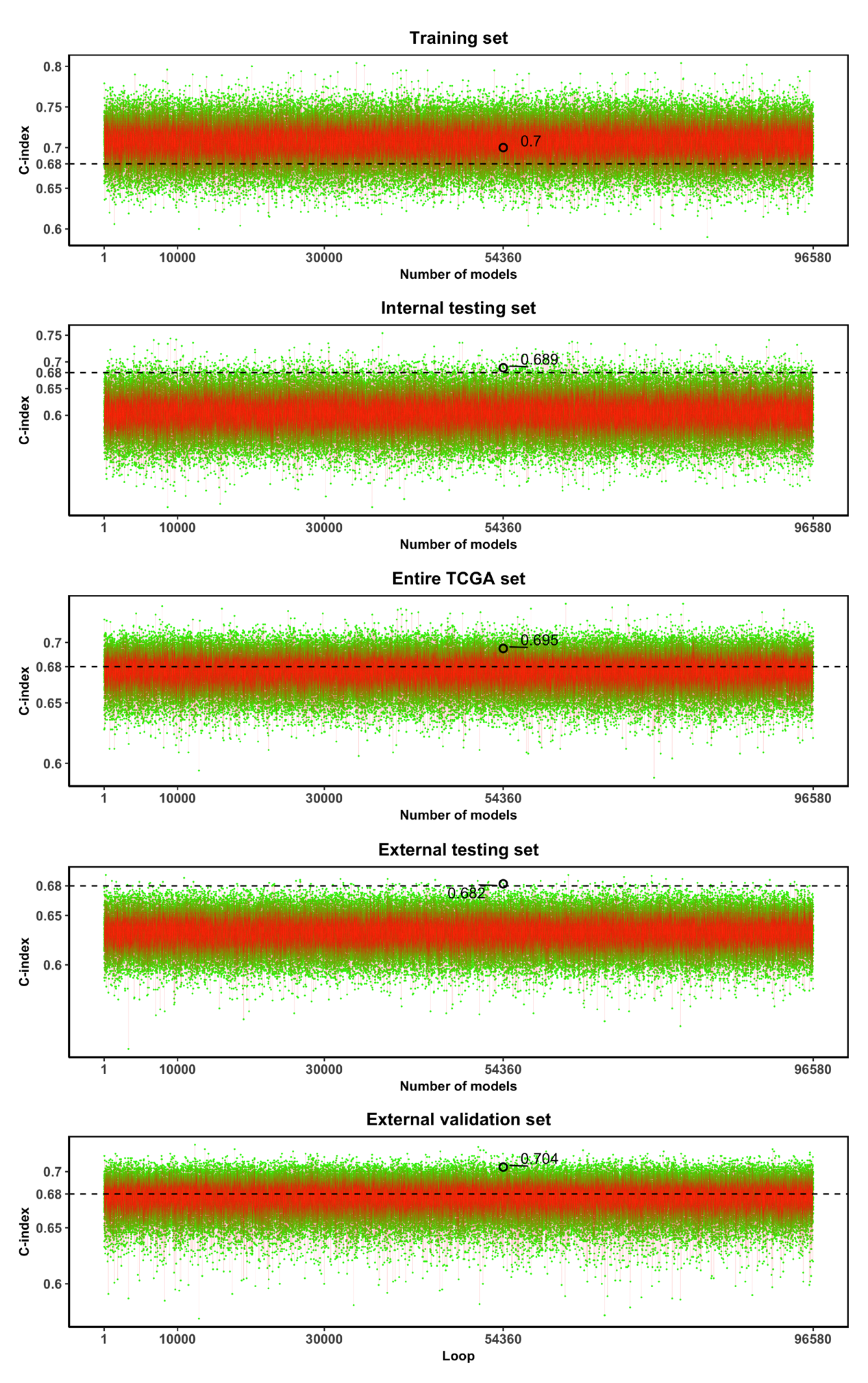


**Figure S2.** Model construction and selection. X-axis represents the number of risk models, y-axis represents the C-index, each green point represents the C-index of a particular risk model. We used red lines to connect these points. The C-indices of the model we captured were highlighted.

**Figure S3.** 16 genes were selected by LASSO regression analysis. A. The dashed vertical line represents the optimal value of log λ with the minimum partial likelihood deviance. B. LASSO coefficient of the 16 genes.

**Figure S4.** Stratification analysis. TCGA stage III LUAD patients were stratified into high- and low- subgroups based the predictive signature, and high-risk group patients had poorer OS compared to low-risk group patients. Hazard Ratio: 2.89; 95% CI: 1.47 − 5.69; p-value < 0.001.

**Figure S5.** Kaplan-Meier survival curves of recurrence-free survival (RFS) between high-risk and low-risk patients. A. TCGA LUAD patients. B. GEO LUAD patients. In both patient datasets, the RFS time of patients in the high-risk group was significantly shorter than that in the low-risk group.

 **Figure S6.** Independent validation on GSE72094 dataset. Kaplan-Meier survival analysis showing the predictive signature can separate GSE72094 LUAD patients into high- and low-risk groups with different OS (*p*-value < 0.0001).
